# Antiviral Effects of Secondary Metabolites from *Jatropha podagrica* Leaves against the Pseudotyped Virus of SARS-CoV-2 Omicron

**DOI:** 10.3390/plants12233942

**Published:** 2023-11-23

**Authors:** Yoon Seo Jang, Da Eun Lee, Dong U Ju, Se Yun Jeong, Yoon-Joo Ko, Changhyun Pang, Ki Sung Kang, Hui-Jeong Gwon, Hee Min Yoo, Ki Hyun Kim

**Affiliations:** 1 School of Pharmacy, Sungkyunkwan University, Suwon 16419, Republic of Korea; bbj0423@gmail.com (Y.S.J.); allag8201@naver.com (D.E.L.); dlawktkark@naver.com (S.Y.J.); 2 Biometrology Group, Korea Research Institute of Standards and Science (KRISS), Daejeon 34113, Republic of Korea; dongwoo0420@korea.ac.kr; 3 School of Biomedical Engineering, Korea University, Seoul 02841, Republic of Korea; 4 Laboratory of Nuclear Magnetic Resonance, National Center for Inter-University Research Facilities (NCIRF), Seoul National University, Gwanak-gu, Seoul 08826, Republic of Korea; yjko@snu.ac.kr; 5 School of Chemical Engineering, Sungkyunkwan University, Suwon 16419, Republic of Korea; chpang@skku.edu; 6 College of Korean Medicine, Gachon University, Seongnam 13120, Republic of Korea; kkang@gachon.ac.kr; 7 Advanced Radiation Technology Institute, Korea Atomic Energy Research Institute, Jeongeup 56212, Republic of Korea; hjgwon@kaeri.re.kr; 8 Department of Precision Measurement, University of Science and Technology (UST), Daejeon 34113, Republic of Korea

**Keywords:** *Jatropha podagrica*, secondary metabolites, chiral HPLC column, antiviral effects, SARS-CoV-2

## Abstract

*Jatropha podagrica* holds a longstanding place in traditional herbal medicine, primarily utilized for addressing skin infections, acting as antipyretics, diuretics, and purgatives. In this study, our primary objective was to investigate the secondary metabolites present in *J. podagrica* leaves, with the aim of pinpointing natural compounds exhibiting potential antiviral activities. Five secondary metabolites (**1**–**5**), including an auronol glycoside (**1**), two coumarins (**2** and **3**), a chromane (**4**) and a gallotannin (**5**), were isolated from *J. podagrica* leaves. Compound **1** presented as an amalgamation of unseparated mixtures, yet its intricate composition was adroitly unraveled through the strategic deployment of a chiral HPLC column. This tactic yielded the isolation of epimers (+)-**1** and (−)-**1**, ascertained as unreported auronol glycosides. The structures of these novel compounds, (+)-**1** and (−)-**1**, were elucidated to be (2*S*)-hovetrichoside C [(+)-**1**] and (2*R*)-hovetrichoside C [(−)-**1**] through NMR data and HR-ESIMS analyses, enzymatic hydrolysis, and comparison of optical rotation values. Cytotoxicity and antiviral effects were assessed for the isolated compounds ((+)-**1**, (−)-**1** and **2**–**5**), along with compound **1a** (the aglycone of **1**), in the A549 human alveolar basal epithelial cell line. Each compound demonstrated a cell viability of approximately 80% or higher, confirming their non-toxic nature. In the group of compounds, compounds **3–5** demonstrated antiviral effects based on RT-qPCR results, with individual enhancements ranging from approximately 28 to 38%. Remarkably, compound **4** exhibited the most substantial antiviral effect. Utilization of compound **4** to assess immune boosting and anti-inflammatory effects revealed increased levels of STING, RIG-I, NLRP3, and IL-10 along with a decrease in TNF-α and IL-6. Therefore, these findings underscore the potential of these active compounds **3**–**5** not only as therapeutic agents for SARS-CoV-2 but also as new contenders for upcoming pandemics.

## 1. Introduction

Severe acute respiratory syndrome coronavirus 2 (SARS-CoV-2) has rapidly spread worldwide, leading to the unprecedented emergence of coronavirus disease 2019 (COVID-19) on a global scale [1]. The SARS-CoV-2 virus features a spike (S) protein, consisting of two subunits, which plays a key role in receptor binding and cell membrane fusion [2]. The Figure S1 subunit contains a receptor-binding domain (RBD) that recognizes and attaches to the angiotensin-converting enzyme 2 (ACE2) host receptor, while the Figure S2 subunit is responsible for fusing the virus with the host cell membrane [2]. Recent research highlights natural products as a promising source of new bioactive compounds with the potential for therapeutic applications against COVID-19 [3,4]. Recent research highlights *Jatropha podagrica* as a promising source of new bioactive compounds with potential therapeutic applications [5].

*Jatropha podagrica*, known by various names such as coral nut, Guatemala rhubarb, and physic nut, is a member of the Euphorbiaceae family. This botanical species is widely distributed across tropical regions in America, Africa and Asia [5,6,7]. With a dual identity as both an ornamental plant and a cornerstone of traditional medicine, *J. podagrica* has been harnessed for a plethora of applications, encompassing pain alleviation, combatting skin infections, addressing sexually transmitted afflictions like gonorrhea, ameliorating jaundice, and fever reduction [8,9,10]. The annals of pharmacological investigation unveil a rich tapestry of attributes ascribed to *J. podagrica* extracts, spanning antifungal, antiproliferative, antioxidant, antitumor, and antibacterial facets [11,12]. In the realm of bioactive phytochemicals, the explorations into *J. podagrica* have unearthed a treasury of compounds: aliphatic acids, coumarins, phenolic acids, steroids, flavonoids, peptides, and diterpenoids, all constituting this vibrant repertoire [11,13,14,15,16,17]. Among these compounds, the noteworthy inclusion of japodic acid, a product of isolation from this botanical marvel, stands out for its insect growth inhibitory potential. Moreover, the ensemble of fraxidin, fraxetin, tomentin, gallic acid, and methyl gallate has donned the mantle of antioxidant and antibacterial agents with notable growth inhibitory capabilities [13,14]. Furthermore, a previous study highlighted that lathyrane diterpenoids isolated from *J. podagrica* exhibited the anti-hepatitis C virus activity [18]. In light of these formidable precedents, the allure of harnessing the manifold potential of bioactive secondary metabolites sourced from *J. podagrica* is irresistibly enticing.

As part of our ongoing pursuit to unveil novel bioactive secondary metabolites and illuminate their structures from a diverse array of natural resources [19,20,21,22,23,24,25], we explored the potential bioactivity intrinsic to secondary metabolites from a methanol (MeOH) extract derived from *J. podagrica* leaves. This endeavor was conducted through a meticulous choreography of liquid chromatography–mass spectrometry (LC/MS) guided analysis, leading us to the isolation of five secondary metabolites (**1**–**5**). Compound **1** posed an initial challenge, presenting itself as an unseparated mixture. However, our endeavor proved triumphant as we employed the dexterity of a chiral high-performance liquid chromatography (HPLC) column to unravel this complexity, which yielded the isolation of epimers (+)-**1** and (−)-**1**, as unreported auronol glycosides. The structures of the new compounds, (+)-**1** and (−)-**1**, were elucidated through an exhaustive analysis of NMR and HR-ESIMS data, as well as optical rotation value. The absolute configuration of the sugar unit was unveiled through a process of chemical transformation followed by enzymatic hydrolysis. Here, we describe the isolation and structural characterization of compounds **1**–**5**, and the evaluation of their antiviral effects against pseudotyped viruses (PVs) of the SARS-CoV-2 Omicron variant.

## 2. Results and Discussion

### 2.1. Isolation and Identification of Compounds ***1–5***

**Figure 1:**
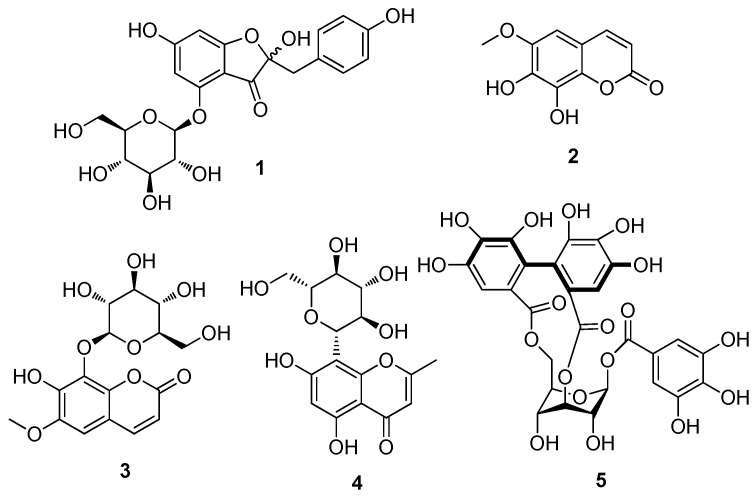
Chemical structures of compounds **1**–**5**.

The extraction process commenced with the partitioning of *J. podagrica* leaf methanol (MeOH) extract between water and organic solvents (hexane, EtOAc, and *n*-BuOH), progressing from lower to higher polarity. This orchestrated maneuver yielded a triumvirate of fractions. Our focal point, the *n*-BuOH soluble fractions arising from this solvent partition, became the epicenter of LC/MS-guided chemical exploration. Through the meticulous orchestration of solvent-partitioned derivation, a symphony of chromatographic processes was conducted over Sephadex LH-20, further enriched by preparative and semi-preparative high-performance liquid chromatography (HPLC) phases. The culmination of these strategic steps resulted in the isolation of one auronol glycoside (**1**), two coumarins (**2** and **3**), a chromane (**4**), and a gallotannin (**5**). The isolated compounds were identified as hovetrichoside C (**1**) [26], fraxetin (**2**) [27], fraxin (**3**) [28], isobiflorin (**4**) [29], and corillagin (**5**) [30] (Figure 1). Their identification was based on a comparison of their NMR spectroscopic data (Figures S3, S7, S9, S11, and S13) with reported values, as well as the results of LC/MS analysis (Figures S2, S6, S8, S10, and S12). To the best of our knowledge, except for **2**, the mentioned compounds were isolated for the first time from *J. podagrica*.

### 2.2. Structural Elucidation of Unreported Auronol Glycoside

**Figure 2:**
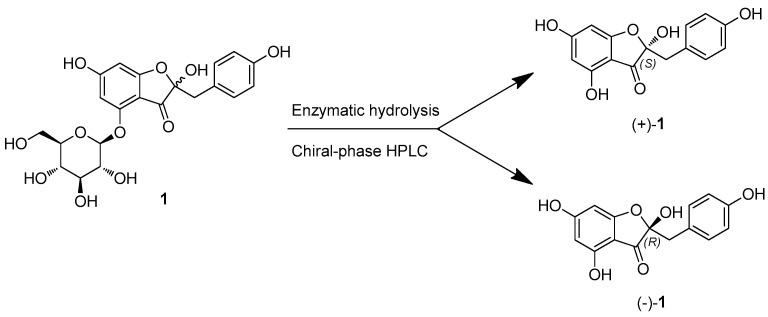
Chiral-phase HPLC purification of Compound **1** and enzymatic hydrolysis for aglycones of (+)-**1** and (−)-**1**.

On the other hand, isolated compound (**1**) was identified as an unseparated mixture since its optical rotation value was almost zero during measurement, which was intended to determine the absolute configuration of compound **1**. This unseparated nature was also confirmed by UPLC-Q-TOF-MS, which exhibited one peak with the molecular formula deduced from the molecular ion peaks: [M-H]^−^ at *m*/*z* 449.1093 (calculated for C_21_H_21_O_11_, 449.1084) in the negative-ion mode. In order to investigate the structures of the isomer mixtures of compound **1**, attempts were made to separate them using a chiral-phase HPLC column. Subsequently, isomers (+)-**1** and (−)-**1** were successfully isolated through chiral-phase HPLC resolution, employing the Daicel CHIRALPAK^®^ OZ-3 LC column (250 × 4.6 mm i.d., 3 μm, Daicel Corporation, Osaka, Japan) (Figure 2). Elution was carried out with hexane-iPrOH (0.1% formic acid) in a 6:4 ratio, at a flow rate of 0.5 mL/min, resulting in the yield of compounds (+)-**1** (*t_R_* 30.0 min, 1.1 mg) and (−)-**1** (*t_R_* 39.0 min, 1.0 mg) in an approximate 1:1 ratio (Figure S1).

The absolute configuration of the glucose units in (+)-**1** and (−)-**1** was assigned using an LC/MS-UV-based method [31,32]. This was further confirmed by enzymatic hydrolysis of (+)-**1** and (−)-**1** using glucosidase, which yielded respective glucopyranoses. The identification of D-glucopyranose was established by comparing the retention time of its thiocarbamoyl–thiazolidine derivative with that of a standard D-glucopyranose sample [33]. The optical rotation values of the aglycones for (+)-**1** and (−)-**1** (${\left[a\right]}_{D}^{25}$ +8.0 for (+)-**1a** and ${\left[a\right]}_{D}^{25}$ −8.0 for (−)-**1a**) indicated a complete separation and an opposite relationship between (+)-**1** and (−)-**1**. A survey of the literature revealed that compounds (+)-**1** and (−)-**1** were identified as previously unreported auronol glycosides, which were determined to be (2*S*)-hovetrichoside C [(+)-**1**] and (2*R*)-hovetrichoside C [(−)-**1**] by 2D NMR data (Figures S4 and S5) and comparison of optical rotation values [34].

### 2.3. Antiviral Activities of Compounds against the Pseudotyped Virus of SARS-CoV-2 Omicron

SARS-CoV-2 pseudotyped viruses (PVs) play an indispensable role in the safe and comprehensive exploration of the virus, serving as a critical tool in various aspects of virology and biomedical research. These PVs provide researchers with the opportunity to study the virus under enhanced biosafety conditions, making it feasible to conduct research in laboratories without high containment facilities. By overcoming accessibility limitations, especially in regions with restricted resources or stringent biosafety regulations, PVs enable broader distribution and facilitate research across diverse locations [35,36,37,38,39,40]. The adaptability of PVs is achieved through the engineering of specific variants of the SARS-CoV-2 spike protein, including variants of concern. This adaptability empowers researchers to delve into the impact of different spike protein mutations on virus–host interactions and immune responses. Additionally, PVs play a crucial role in target cell studies, enabling investigations into viral entry mechanisms and the evaluation of potential neutralization strategies such as convalescent plasma and monoclonal antibodies [41,42,43]. In the context of antiviral drug development, SARS-CoV-2 PVs serve as high-throughput platforms for screening potential therapeutic compounds, expediting the discovery of effective agents [44]. Within the field of vaccine development, PVs provide valuable tools for studying viral entry mechanisms and assessing the efficacy of candidate vaccines. This safer alternative for vaccine testing contributes significantly to the development and evaluation of SARS-CoV-2 vaccines. 

**Figure 3:**
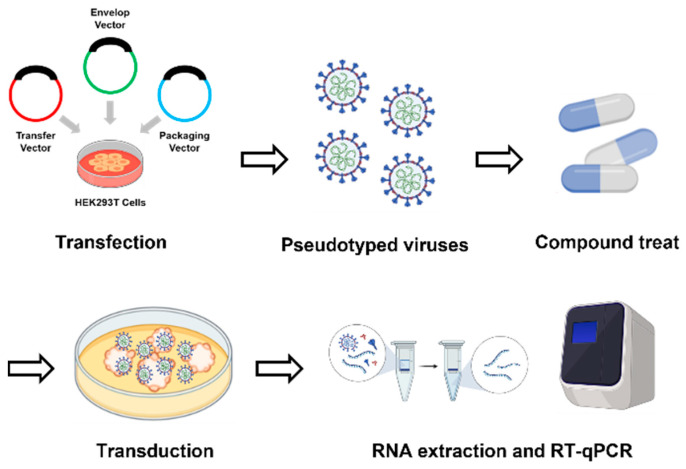
A schematic overview of the screening of compounds for antiviral effect.

Numerous methodologies detailing the steps to generate SARS-CoV-2 pseudotyped viruses (PVs) have been made publicly available worldwide [45,46,47,48]. In our study, we employed the following approach to create PVs. We prepared a mixture of either GFP or GFP-fused Spike protein from the Omicron variant, along with an envelope and packaging vector. This mixture of plasmids was introduced into cells using Metafectene PRO (Biontex, Munich, Germany) for transfection. Subsequently, viral supernatant was collected at either 48 or 72 h intervals post transfection and stored at −80 °C (Figure 3).

**Figure 4:**
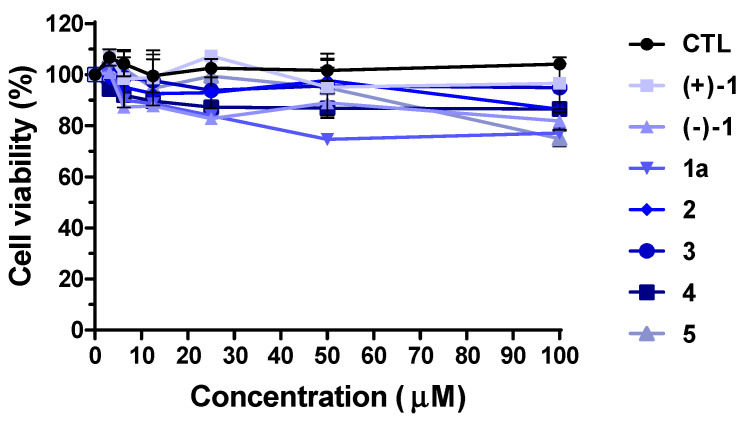
Cell viability of compounds **1**–**5** (100 μM) or DMSO control (CTL) on A549 human alveolar basal epithelial cells.

Using the pseudotyped viruses generated through the process, we evaluated the antiviral effects of the isolated compounds ((+)-**1**, (−)-**1**, and **2**–**5**), along with compound **1a** (the aglycone of (+)-**1**). Before conducting the antiviral assessment, we performed an MTS assay on the A549 cell line to determine the potential toxicity of the compounds. The results demonstrated that all compounds ((+)-**1**, (−)-**1**, **1a**, and **2**–**5**) exhibited cell viability exceeding 80% (Figure 4). The drug dose used in the MTS assay has been employed in various other natural compound experiments [49,50,51,52,53,54], and the confirmed dosage range falls within the working range commonly used in antiviral experiments. Based on our results, antiviral activity was demonstrated at lower concentrations (10 μM), while higher concentrations (100 μM) are sufficient to validate toxicity. This indicated that none of the compounds displayed cytotoxic effects, thus enabling us to proceed with subsequent experiments.

**Figure 5:**
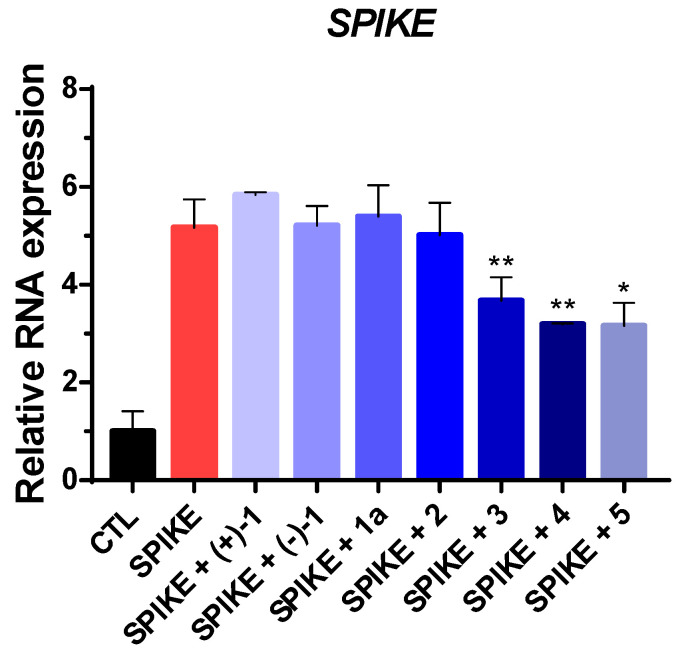
Infectivity on compounds (+)-**1**, (−)-**1**, **1a**, and **2**–**5** (10 µM) or DMSO control (CTL) using pseudotyped virus (PV) of SARS-CoV-2 Omicron variant. Values indicate means ± SEM (*n* = 3, * *p* < 0.05, ** *p* < 0.01 vs. control group).

Following the confirmation of the compounds’ non-toxic nature through the MTS assay, their antiviral effects were evaluated using the A549 cell line. In each well, the compounds (10 μM) were treated alongside PVs in equal amounts. After 36 h, viral RNA was extracted from the infected cells for analysis. Quantitative assessment of the virus was conducted using RT-qPCR targeting the spike gene. The results of the analysis reveal a notable reduction in compounds **3**–**5** when compared to the treatment with SARS-CoV-2 Omicron PVs alone. Specifically, compound **3** demonstrated a reduction of approximately 28%, while compounds **4** and **5** exhibited even greater reductions of around 38% in comparison to the effects of SARS-CoV-2 Omicron PVs alone (Figure 5). These findings conclusively indicate that compounds **3**–**5** effectively contribute to diminishing the viral load within the SARS-CoV-2 Omicron PV-infected cell model.

Among the isolated compounds from *J. podagrica* used in this experiment, compounds **3**–**5** correspond to coumarin, chromone, and tannin, respectively. The effects of each compound on viral activity were investigated, and their impacts were assessed considering the experimental results. The study also identified areas that require further research. Coumarin, chromone, and tannin used in the experiment exhibit the following properties: Coumarins are widely distributed, stable, and soluble, low-molecular-weight compounds that can be chemically modified to produce new semisynthetic derivatives [55,56]. They are of great interest in medicinal chemistry due to their lack of adverse side effects and toxicity [57]. Thus, coumarins have received considerable attention as promising candidates for anti-viral drugs, targeting various cellular pathways that inhibit the growth and replication of viruses [58]. Possible approaches for treating COVID-19 using natural coumarins involve interfering with various stages of the viral life cycle, such as the binding of the virion to the receptor present on the cell surface [55]. It is important to consider the potential of such compounds as a therapeutic intervention for the management of COVID-19 [55]. Furthermore, their antiviral properties are of great interest for the development of effective medication against viral infections [55,56,57,58]. During this stage, there is an excess of soluble forms of angiotensin-converting enzyme 2 (ACE2) involved [59]. Therefore, the use of ACE2 inhibitors is a potential strategy in treating COVID-19 [60]. Chromones are important components in natural products, pharmaceuticals, and bioactive molecules, which makes them an important part of human nutrition [61,62,63]. According to molecular docking studies, chromones exhibit the potential to inhibit the RBD function of the SARS-CoV-2-S protein [64]. Moreover, chromones act as mast cell (MC) stabilizers, which can alleviate respiratory complications linked to SARS-CoV-2 infections [65]. The response of the host to virus invasions through RNA activation is complex and can result in both positive and detrimental effects [64,65]. Immune cells including MCs situated in the submucosa of the respiratory tract promptly target Coronaviruses [66]. MCs activated through Toll-like receptor-3 (TLR3) produce interferon (IFN) α, β and IL-8, and recruit NK cells [67]. Since the outset of the COVID-19 pandemic, much research has focused on preventing RBD/ACE2 binding [2], while natural polyphenols may additionally target two proteolytic enzymes: the transmembrane protease serine 2 (TMPRSS2) and the intracellular 3-chymotrypsin-like cysteine protease (3CLpro). In previous studies, tannic acid has been recognized as a powerful suppressant of both TMPRSS2 and 3CLpro [68]. In addition, tannic acid has shown potent inhibition of main protease (Mpro)/3CLpro and TMPRSS2, which are essential proteins for the propagation of SARS-CoV-2 [69,70]. In particular, it was confirmed that tannic acid prevented pseudotyped SARS-CoV-2 entrance by preventing S-RBD from attaching to ACE2 [71,72]. Notably, orally administered tannic acid isomers have exhibited effectiveness against SARS-CoV-2 variants such as Delta and Omicron [73]. 

Therefore, the treatment with the isolated compounds from *J. podagrica*, specifically compounds **3**–**5**, has shown an approximately two-fold reduction compared to the PV-infected control, resulting in an enhanced antiviral effect as PV of SARS-CoV-2 inhibitors. Furthermore, by inhibiting ACE2, the active compounds from *J. podagrica* exhibit substantial potential as antiviral agents against the actual SARS-CoV-2 and future pandemics. Further research is needed to validate this potential through in vitro and in vivo studies and to assess its applicability in real viruses, not just pseudotyped viruses. 

Pseudoviruses (PVs) serve as advantageous tools in numerous scientific investigations due to the benefits associated with their application in research. The study of viral entry processes and assessments for antiviral drug development could potentially be made safer, better controlled, and more accessible with the use of pseudoviruses. However, while pseudoviruses offer certain advantages, they come with several limitations [74]. Firstly, pseudoviruses are only applicable in the study of viruses that possess specific envelope proteins, such as influenza virus, coronavirus, retrovirus, and herpesvirus [75]. Secondly, the characteristics emulated by pseudoviruses are considerably constrained when compared to live viruses. Primarily, pseudoviruses can replicate the role of the live virus envelope protein in facilitating viral entry into cells in vitro. However, they cannot replicate the subsequent intracellular processes of viral proliferation and release. Consequently, it is essential to compare and validate results obtained from assays using pseudotyped viruses against live virus-based assays, which continue to be the gold standard [76].

### 2.4. The Immune Regulator of Compound ***4*** Is Crucial in Modulating the Immune Response and Inflammation

The cGAS-STING (cyclic gaunosine monophosphate (GMP) adenosine monophosphate (*AMP*) Synthase-Stimulator of interferon genes) pathway plays a crucial role in the intracellular immune response to viral infections, particularly in the detection of viral DNA and activation of interferon genes [77,78,79]. The Retinoic Acid-Inducible Gene I (*RIG* I) pathway also plays a vital role in the intracellular immune response to viral infections, specifically in detecting viral RNA and activating interferon genes [80,81]. Moreover, NOD-like receptor protein (*NLRP*)-mediated *NLRP3* signaling pathway serves as a protein complex that regulates inflammation and immune responses, detecting various signals within the cell and promoting inflammatory responses. The *NLRP3* inflammasome pathway plays a significant role in regulating the immune system [82,83,84]. In addition, IL-6, IL-1β, and IL-10 are all classified as cytokines, protein components that play a role in regulating the immune system.

**Figure 6:**
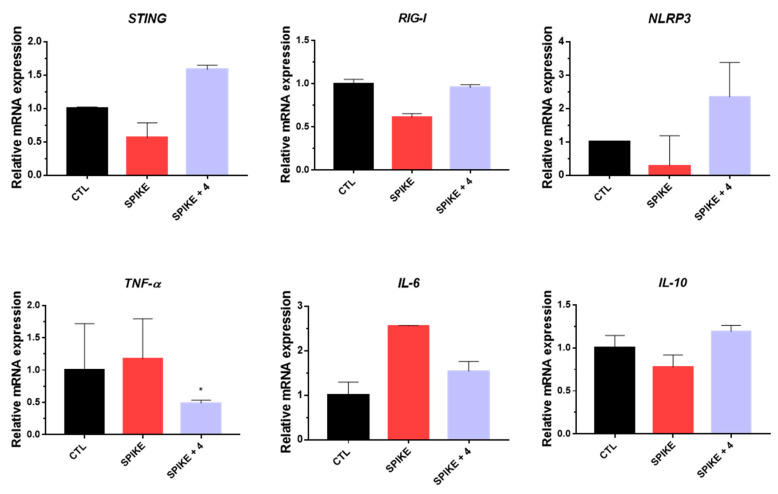
The inhibitory effects of compound **4** on SARS-CoV-2 Omicron variant pseudovirus (PV) infection in A549 cells were evaluated for STING, RIG-I, NLRP3, TNF-α, IL-6, and IL-10 genes. Values indicate means ± SEM (*n* = 3, * *p* < 0.05 vs. control group).

After treatment with compound **4**, an increase in the mRNA expression levels of STING, RIG-1, and NLRP3 genes was observed, while the mRNA expression levels of TNF-α and IL-6 decreased compared to those of the virus-infected group (Figure 6). The study confirmed both immune-boosting effects and the reduction in TNF-α and IL-6 as a means to alleviate inflammation. In inflammatory conditions, the mRNA level of IL-10 often increases, which is in the opposite direction of TNF-α and IL-6. Specifically, after treatment with compound **4**, it was noted that the mRNA level of TNF-α and IL-6 decreased, whereas the mRNA level of IL-10 increased, confirming the anti-inflammatory properties of the natural products derived from compound **4**. Therefore, it was concluded that treatment with compound **4** not only exhibited antiviral effects, but also possessed anti-inflammatory and immune-boosting effects through multiple signaling pathways.

## 3. Materials and Methods

### 3.1. General Experimental Procedure 

Liquid chromatography-mass spectrometry (LC-MS) analysis was performed using an Agilent 1200 series HPLC system with a diode array detector and a 6130 Series ESI mass spectrometer. The analysis utilized an analytical Kinetex C18 100 Å column (100 mm × 2.1 mm i.d., 5 μm; flow rate: 0.3 mL/min) from Phenomenex. All high-resolution electrospray ionization mass spectra (HR-ESIMS) data were obtained employing an Agilent G6545B quadrupole time-of-flight mass spectrometer (Agilent Technologies, Santa Clara, CA, USA) coupled with an Agilent 1290 Infinity II HPLC instrument. The HPLC instrument used an Agilent Eclipse Plus C18 column (2.1 × 50 mm, 1.8 μm; flow rate: 0.3 mL/min). Circular dichroism (CD) spectra were recorded using a JASCO J-810 spectropolarimeter (Jasco, Oklahoma City, OK, USA). Nuclear magnetic resonance (NMR) spectra were acquired using a Bruker AVANCE III instrument (Bruker, Billerica, MA, USA). Medium-pressure liquid chromatography (MPLC) was conducted using the Smart Flash AKROS system (Yamazen, Osaka, Japan) and an analytical Universal ODS-SM 120 Å column (3.0 × 20.0 cm, 50 μm) from Yamazen. Preparative high-performance liquid chromatography (HPLC) was performed utilizing a Waters 1525 binary HPLC pump with a Waters 996 photodiode array detector (Waters Corp.). The system was equipped with a Hector C18-A510025200 column (21.2 × 250 mm, 5 μm; flow rate: 5 mL/min). Semi-preparative HPLC was carried out using a Waters 1525 binary HPLC pump with a Waters 996 photodiode array detector (Waters Corp.). The setup featured a Phenomenex Luna phenyl–hexyl column (250 × 10 mm i.d., flow rate: 2 mL/min). Column chromatography was performed using Sephadex LH-20 (Pharmacia, Uppsala, Sweden) and Diaion HP-20 (Mitsubishi Chemical Co., Ltd., Tokyo, Japan). Thin-layer chromatography (TLC) was conducted using precoated silica gel F254 plates and RP-18 F254s plates from Merck. TLC spots were detected under UV light after being dipped in anisaldehyde–sulfuric acid and heated.

### 3.2. Plant Materials

*J. podagrica* stalks and roots were collected from Thanh Hoa province, Vietnam, in August 2020, and subsequently dried. The plant’s identification was authenticated by the corresponding author (K.S.K.). To ensure accurate identification, a specimen (SHYD-2020-08) was preserved at the herbarium of the School of Pharmacy, Sungkyunkwan University, Suwon, Republic of Korea.

### 3.3. Extraction and Isolation 

The air-dried stalks and roots of *J. podagrica* (400 g) were subjected to extraction three times using an 80% aqueous MeOH (2.1 L × 3 days each) at 70 °C with a reflux condenser. The extracts were then filtered, and the filtrate was concentrated using a rotavapor to yield the MeOH extract (272 g). This extract was suspended in 700 mL of distilled water and underwent solvent fractionation in triplicate, utilizing 700 mL of hexane, ethyl acetate, and *n*-butanol, respectively. After concentration, three fractions with varying polarities were obtained: the hexane-soluble fraction (20.6 g), the ethyl acetate-soluble fraction (10.4 g), and the *n*-butanol-soluble fraction (10.4 g). To eliminate the sticky sugar residue from the *n*-butanol fraction, an HP-20 open column chromatography was conducted, using 6 L of distilled water, methanol, and acetone as eluents. After concentration, three fractions were obtained: the water fraction (6.3 g), the methanol fraction (3.9 g), and the acetone fraction (0.1 g). The methanol fraction (3.9 g) was further subjected to medium pressure liquid chromatography (MPLC) and eluted with a gradient solvent system of MeOH/H_2_O (90:10 to 50:50), resulting in the isolation of seven fractions (A–G). Fraction D (580 mg) from this process underwent open column chromatography using a Sephadex LH-20 column. The mobile phase started with the MeOH/H_2_O mixture (80:20) and transitioned to a 100% MeOH. This yielded eight subfractions (D1–D8). Subfraction D4 (53.6 mg) was purified (29% MeOH/H_2_O), resulting in the isolation of compounds **1** (3.7 mg, *t*_R_ = 42.0 min, ESIMS *m*/*z* 473 [M+Na]^+^), **3** (4.5 mg, *t*_R_ = 37.5 min, ESIMS *m*/*z* 393 [M+Na]^+^), and **4** (1.7 mg, *t*_R_ = 27.0 min, ESIMS *m*/*z* 355 [M+H]^+^). Furthermore, subfraction D6 (25.4 mg) underwent purification via semi-preparative HPLC, eluted with a 39% MeOH/H_2_O, leading to the isolation of compound **2** (4.6 mg, *t*_R_ = 25.5 min, ESIMS *m*/*z* 209 [M+H]^+^). Compound **5** (3.4 mg, *t*_R_ = 21.5 min, ESIMS *m*/*z* 633 [M-H]^−^) was also purified from subfraction D7 (15.8 mg) using semi-preparative HPLC (33% MeOH/H_2_O).

### 3.4. Acid Hydrolysis and Absolute Configuration Determination of the Sugar Moiety of ***1***

Compounds (+)-**1** and (−)-**1** (each 0.5 mg) were hydrolyzed using glucosidase (10 mg, from almonds, Sigma-Aldrich, St. Louis, MO, USA) for 72 h at 37 °C, and CH_2_Cl_2_ was used for the extraction of aglycone. The aqueous layer was neutralized by repeated evaporation using a vacuum evaporator and dissolved in anhydrous pyridine (0.5 mL) with the addition of L-cysteine methyl ester hydrochloride (0.7 mg). After the reaction mixture was heated at 60 °C for 1 h, *o*-tolylisothiocyanate (50 μL) was added, and the mixture was kept at 60 °C for 1 h. The reaction product was evaporated using a vacuum evaporator and dissolved in MeOH. The dissolved reaction product then was directly analyzed by LC/MS [0% MeOH → 70% MeOH gradient system (0–30 min), 70% MeOH → 100% MeOH (30–31 min), 100% MeOH (31–41 min), 0% MeOH (42–52 min), and 0.3 mL/min] using an analytical Kinetex C18 100 Å column (100 mm × 2.1 mm i.d., 5 μm). The sugar moiety from (+)-**1** and (−)-**1** was identified as D-glucopyranose based on a comparison of the retention time of an authentic sample (*t*_R_: D-glucopyranose 13.9 min).

### 3.5. MTS Assay

The impact of compounds on the growth of A549 cells was evaluated. Initially, 10,000 cells were placed in each well of 96-well plates and allowed incubation overnight. Subsequently, the cells were exposed to varying concentrations of the isolated compounds for a 48 h period. Following this exposure, a solution containing 3-(4,5-dimethylthiazol-2-yl)-5-(3-carboxymethoxyphenyl)-2-(4-sulfophenyl)-2*H*-tetrazolium (MTS) was introduced into each well, and the cells were incubated at a temperature of 37 °C in a 5% CO_2_ environment for 4 h. The assessment of cell viability was performed by measuring the absorbance of the resulting solution at 490 nm using a Synergy HTX Multi-Mode microplate reader (BioTek Instruments, Inc., Winooski, VT, USA). 

### 3.6. RT-qPCR Analysis

The RT-qPCR analysis was executed using the StepOne and StepOnePlus Real-Time PCR systems from Thermo Fisher Scientific, Waltham, MA, USA in combination with the One Step PrimeScript RT-PCR Kit (Perfect Real Time) or Ex taq provided by Takara (Takara Bio Inc., Shiga, Japan). The total reaction volume for the RT-qPCR was 20 μL, and the reaction mixture was meticulously prepared following the manufacturer’s precise instructions.

### 3.7. Statistical Analysis

Statistics were analyzed with GraphPad Prism (GraphPad Software, Inc., San Diego, CA, USA). Student’s *t*-test was used for further data analysis. *p* values * *p* < 0.05 and ** *p* < 0.01 were considered statistically significant.

## 4. Conclusions

In summary, this study focused on the phytochemical analysis of MeOH extracts from *J. podagrica* leaves, leading to the identification of five secondary metabolites: hovetrichoside C (**1**), fraxetin (**2**), fraxin (**3**), isobiflorin (**4**), and corillagin (**5**). Compound **1** was initially found in the form of unseparated mixtures, which were successfully separated using a chiral HPLC column. This separation yielded epimers (+)-**1** and (−)-**1**, identified as novel auronol glycosides, which were determined as (2*S*)-hovetrichoside C [(+)-**1**] and (2*R*)-hovetrichoside C [(−)-**1**]. Furthermore, the study investigated the antiviral potential of compounds (+)-**1**, (−)-**1**, **1a**, and **2**–**5** against SARS-CoV-2 Omicron pseudotyped viruses (PVs). Among the seven compounds assessed, compounds **3**–**5** demonstrated the capacity to inhibit PV infection. Importantly, these active compounds did not display any cellular toxicity. The results obtained from RT-qPCR confirm that compounds **3**–**5** exhibited an approximately two-fold increase in their antiviral effects. Concurrently, ongoing studies investigated the immune-boosting and anti-inflammatory capabilities of the most active compound **4** concerning SARS-CoV-2, yielding valuable new insights. These inquiries assume a pivotal role in advancing and validating traditional herbal medicine, particularly the active compounds isolated from *J. podagrica*. Given their potential as candidates for disease control, it is imperative to comprehensively evaluate the biological activity of compounds **3**–**5**. Consequently, further in vivo studies focusing on antiviral effects and anti-inflammation are warranted. In summary, compounds **3**–**5** demonstrated potential as SARS-CoV-2 inhibitors, making them compelling candidates for subsequent in vivo investigations. 

## Supplementary Materials

The following are available online at https://www.mdpi.com/article/10.3390/plants12233942/s1: Figure S1: The chiral phase HPLC chromatography of compound **1**; Figure S2: UV chromatogram of LC/MS, and UV and MS data for compound **1**; Figure S3: The ^1^H NMR spectrum of **1** (CD_3_OD, 850 MHz); Figure S4: The HSQC spectrum of **1** (CD_3_OD); Figure S5: The HMBC spectrum of **1** (CD_3_OD); Figure S6: UV chromatogram of LC/MS, and UV and MS data for compound **2**; Figure S7: The ^1^H NMR spectrum of **2** (CD_3_OD, 850 MHz); Figure S8: UV chromatogram of LC/MS, and UV and MS data for compound **3**; Figure S9: The ^1^H NMR spectrum of **3** (CD_3_OD, 850 MHz); Figure S10: UV chromatogram of LC/MS, and UV and MS data for compound **4**; Figure S11: The ^1^H NMR spectrum of **4** (CD_3_OD, 850 MHz); Figure S12: UV chromatogram of LC/MS, and UV and MS data for compound **5**; Figure S13: The ^1^H NMR spectrum of **5** (CD_3_OD, 850 MHz).
