# Supplementary material for: Antiviral Effects of Secondary Metabolites from Jatropha podagrica Leaves against the Pseudotyped Virus of SARS-CoV-2 Omicron
Source: Plants (Basel). 2023 Nov 23;12(23):3942. doi: 10.3390/plants12233942 (PMC10708114; doi:10.3390/plants12233942)

Supplementary Materials

Antiviral Effects of Secondary Metabolites from *Jatropha podagrica* Leaves against the Pseudotyped Virus of SARS-CoV-2 Omicron

Yoon Seo Jang [1,†], Da Eun Lee [1,†], Dong U Ju [2,3,†], Se Yun Jeong [1], Yoon-Joo Ko [4], Changhyun Pang [5], Ki Sung Kang [6], Hui-Jeong Gwon [7], Hee Min Yoo [2,8,]* and Ki Hyun Kim [1,]*

[1]*School of Pharmacy, Sungkyunkwan University, Suwon 16419, Republic of Korea; bbj0423@gmail.com (Y.S.J.); allag8201@naver.com (D.E.L.); dlawktkark@naver.com (S.Y.J.)*
[2]*Biometrology Group, Korea Research Institute of Standards and Science (KRISS), Daejeon 34113, Republic of Korea; dongwoo0420@korea.ac.kr*
[3]*School of Biomedical Engineering, Korea University, Seoul 02841, Republic of Korea*
[4]*Laboratory of Nuclear Magnetic Resonance, National Center for Inter-University Research Facilities (NCIRF), Seoul National University, Gwanak-gu, Seoul 08826, Republic of Korea; yjko@snu.ac.kr*
[5]*School of Chemical Engineering, Sungkyunkwan University, Suwon 16419, Republic of Korea; chpang@skku.edu*
[6]*College of Korean Medicine, Gachon University, Seongnam 13120, Republic of Korea; kkang@gachon.ac.kr*
[7]*Advanced Radiation Technology Institute, Korea Atomic Energy Research Institute, Jeongeup 56212 Republic of Korea; hjgwon@kaeri.re.kr*
[8]*Department of Precision Measurement, University of Science and Technology (UST), Daejeon 34113, Republic of Korea*
**Correspondence: hmy@kriss.re.kr (H.M.Y.); khkim83@skku.edu (K.H.K.); Tel.: +82-31-290-7700 (K.H.K.)*
[†]*These authors contributed equally to this study.*

Supporting Information Contents:

Figure S1. The chiral phase HPLC chromatography of compound **1**……………………………..……………………………...…………S3
Figure S2. UV chromatogram of LC/MS, and UV and MS data for compound **1**……………………………...………………...……S4
Figure S3. The ^{1}H NMR spectrum of **1** (CD_3OD, 850 MHz) ………………………………………..……………………………………..S5
Figure S4. The HSQC spectrum of **1** (CD_3OD) ……………..…………………………...……………………………………………..S6
Figure S5. The HMBC spectrum of **1** (CD_3OD) ………………...…………………………...………………………………………S7
Figure S6. UV chromatogram of LC/MS, and UV and MS data for compound **2**……………………………………………………………..S8
Figure S7. The ^{1}H NMR spectrum of **2** (CD_3OD, 850 MHz) ……………………………………………………………………………S9
Figure S8. UV chromatogram of LC/MS, and UV and MS data for compound **3**……………………………...…………………………S10
Figure S9. The ^{1}H NMR spectrum of **3** (CD_3OD, 850 MHz) ………………………………...…………………………………………S11
Figure S10. UV chromatogram of LC/MS, and UV and MS data for compound **4**……………………………...……………………...…S12
Figure S11. The ^{1}H NMR spectrum of **4** (CD_3OD, 850 MHz) ……………………………………...……………………………………..S13
Figure S12. UV chromatogram of LC/MS, and UV and MS data for compound **5**……………………………...……………………...…S14
Figure S13. The ^{1}H NMR spectrum of **5** (CD_3OD, 850 MHz) ……………………………………...……………………………...…S15

Figure S1. The chiral phase HPLC chromatography of compound **1**

Column: CHIRALPARK® OZ-3 LC column (250 × 4.6 mm i.d., 3 μm, Daicel Corporation, Osaka, Japan)

Flow rate: 0.5 mL/min

Mobile phase: 60:40:0.1 (Hexanes/*i*ProH/Formic Acid)

Injection: 10 μL

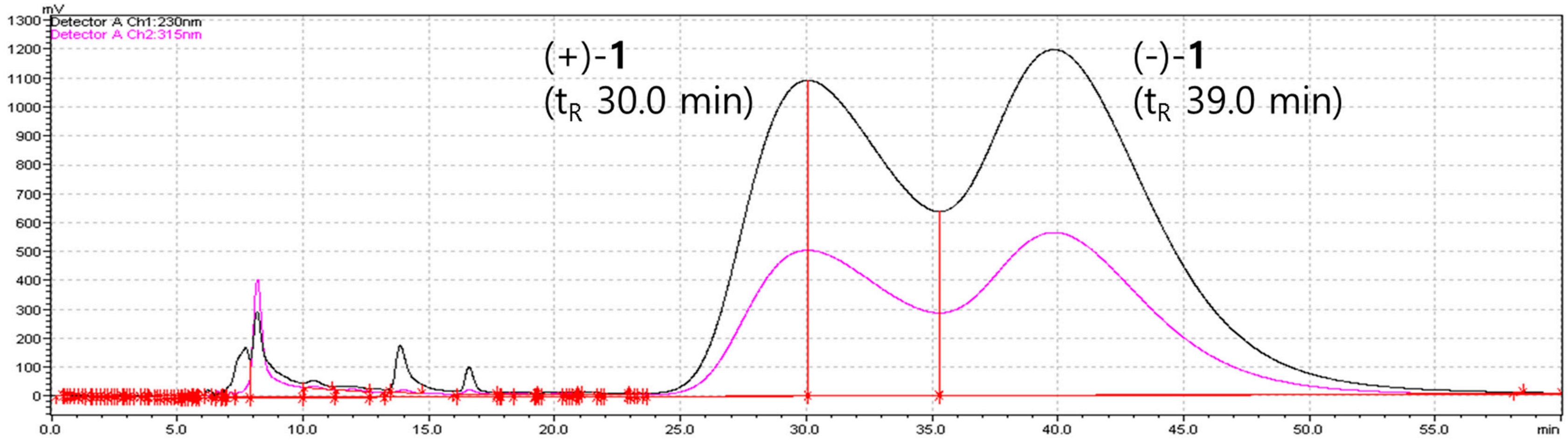

Figure S2. UV chromatogram of LC/MS, and UV and MS data for compound **1**

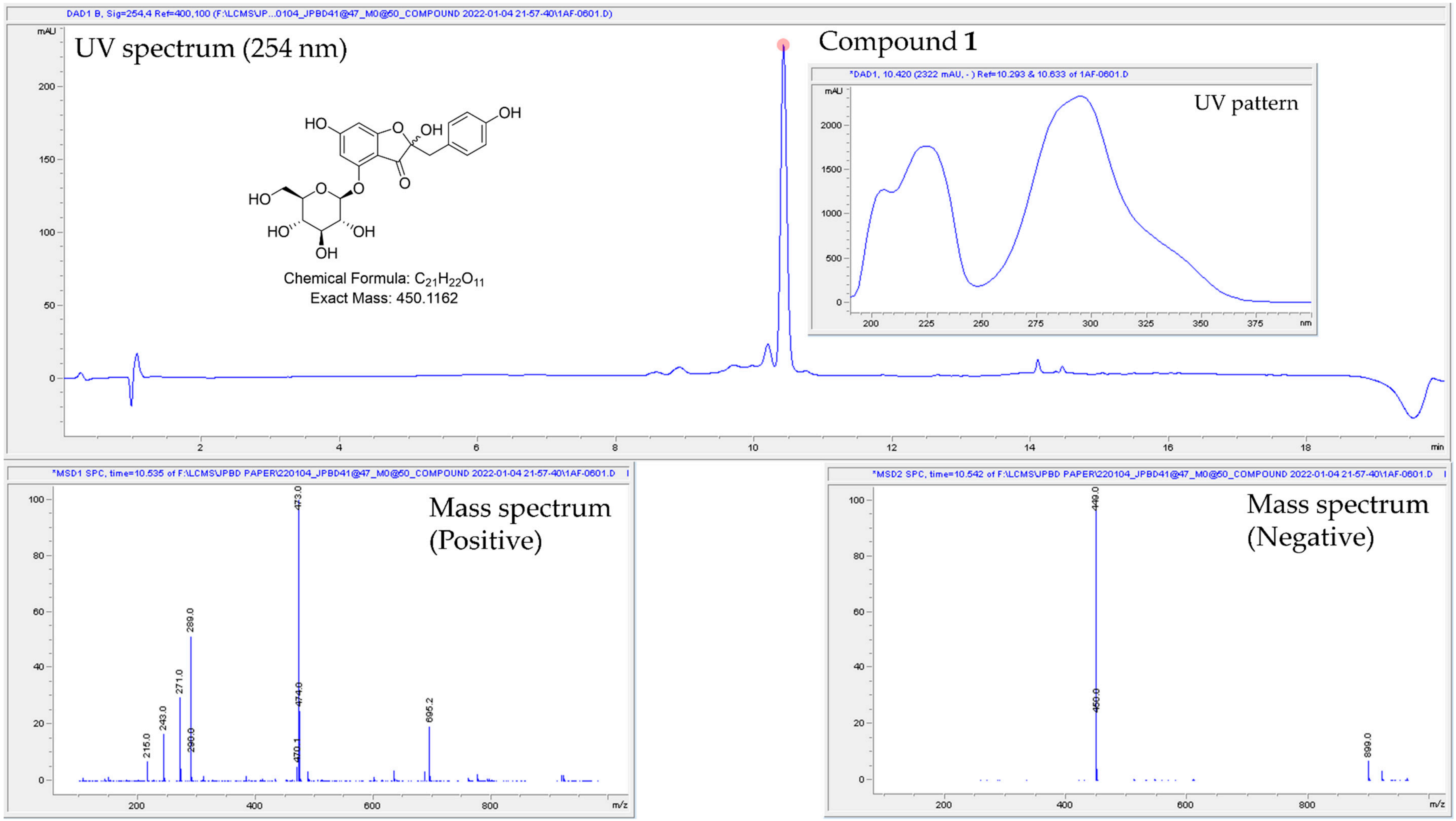

Figure S3. The ^{1}H NMR spectrum of **1** (CD_3OD, 850 MHz)

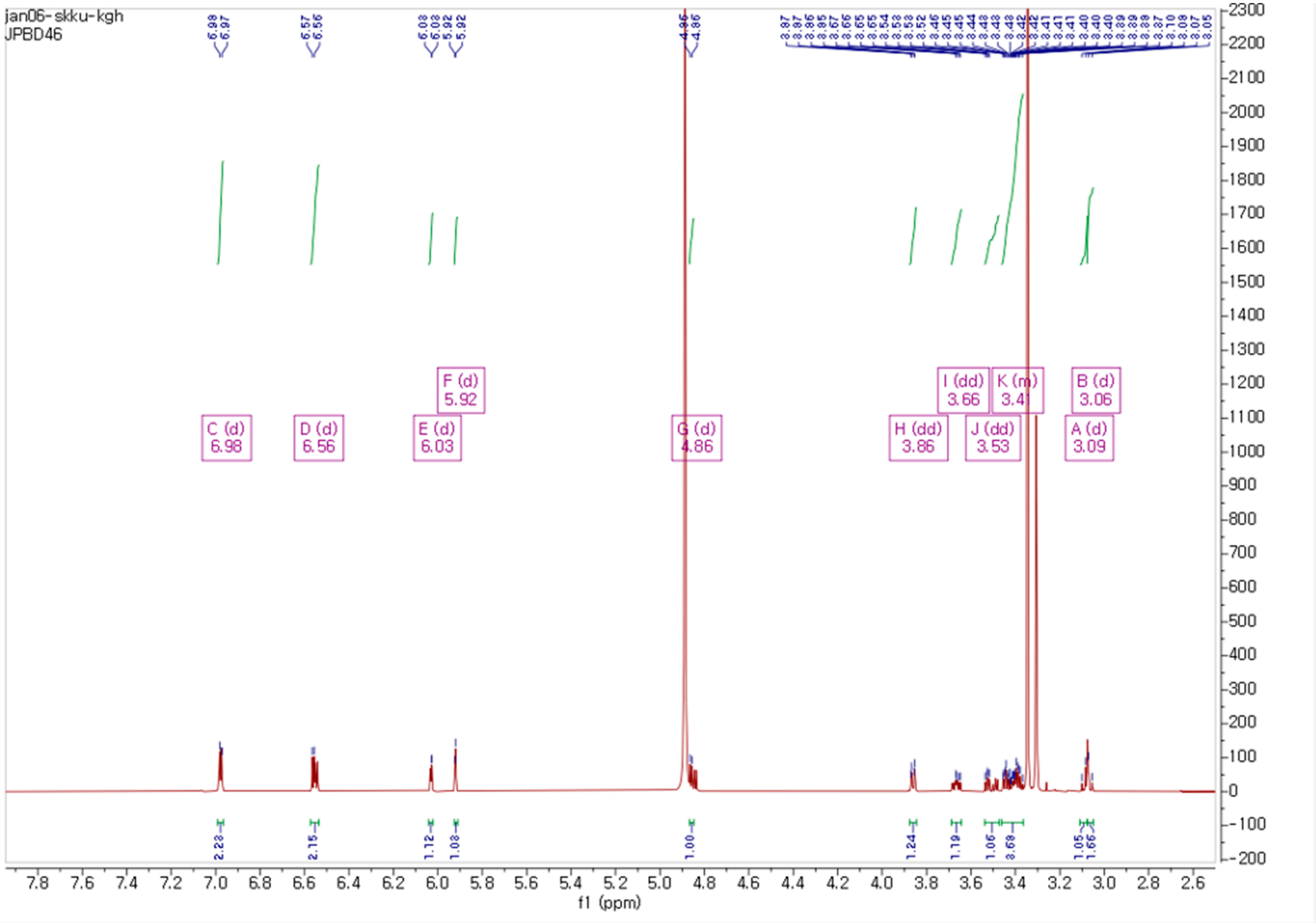

Figure S4. The HSQC spectrum of **1** (CD_3OD)

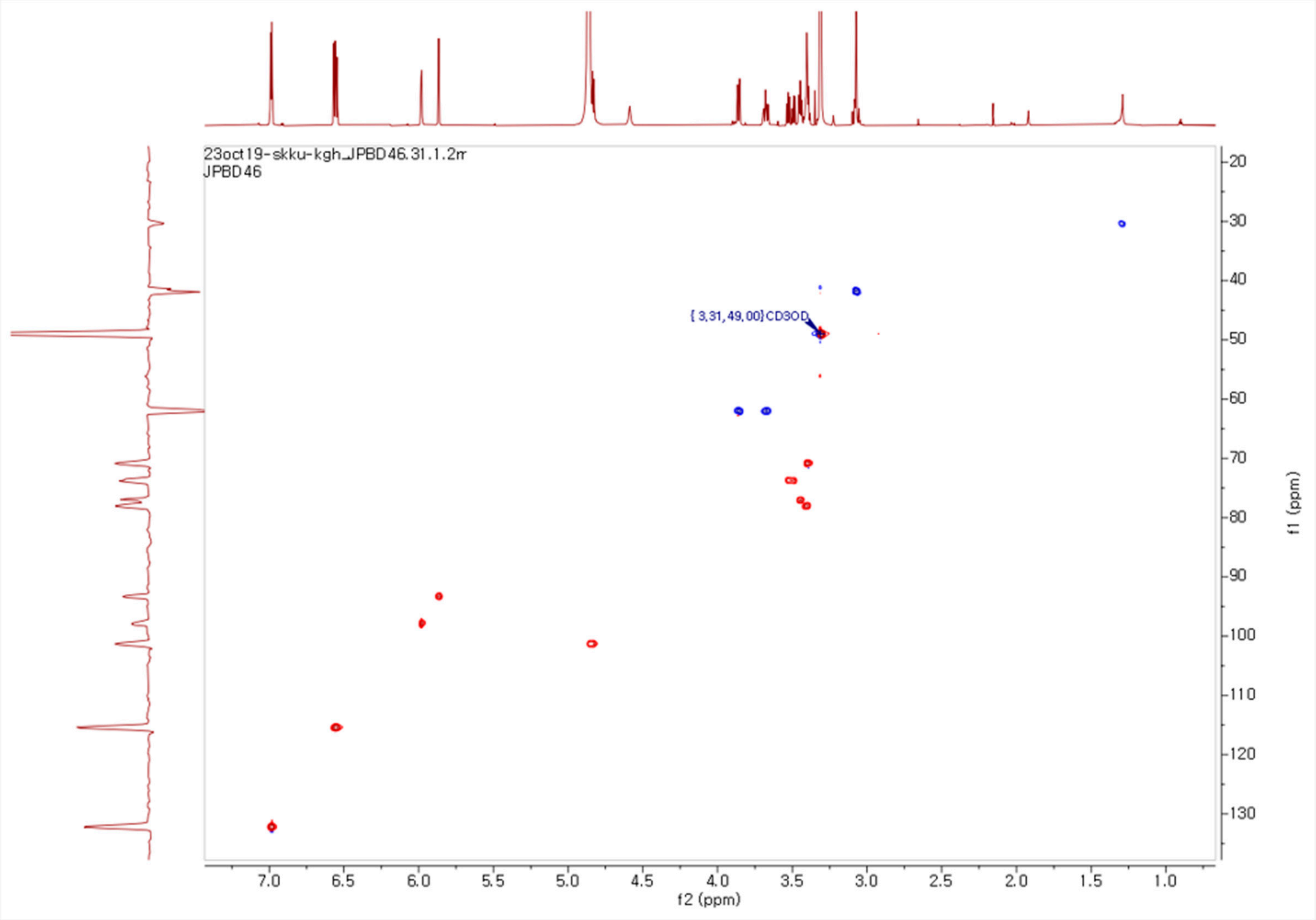

Figure S5. The HMBC spectrum of **1** (CD_3OD)

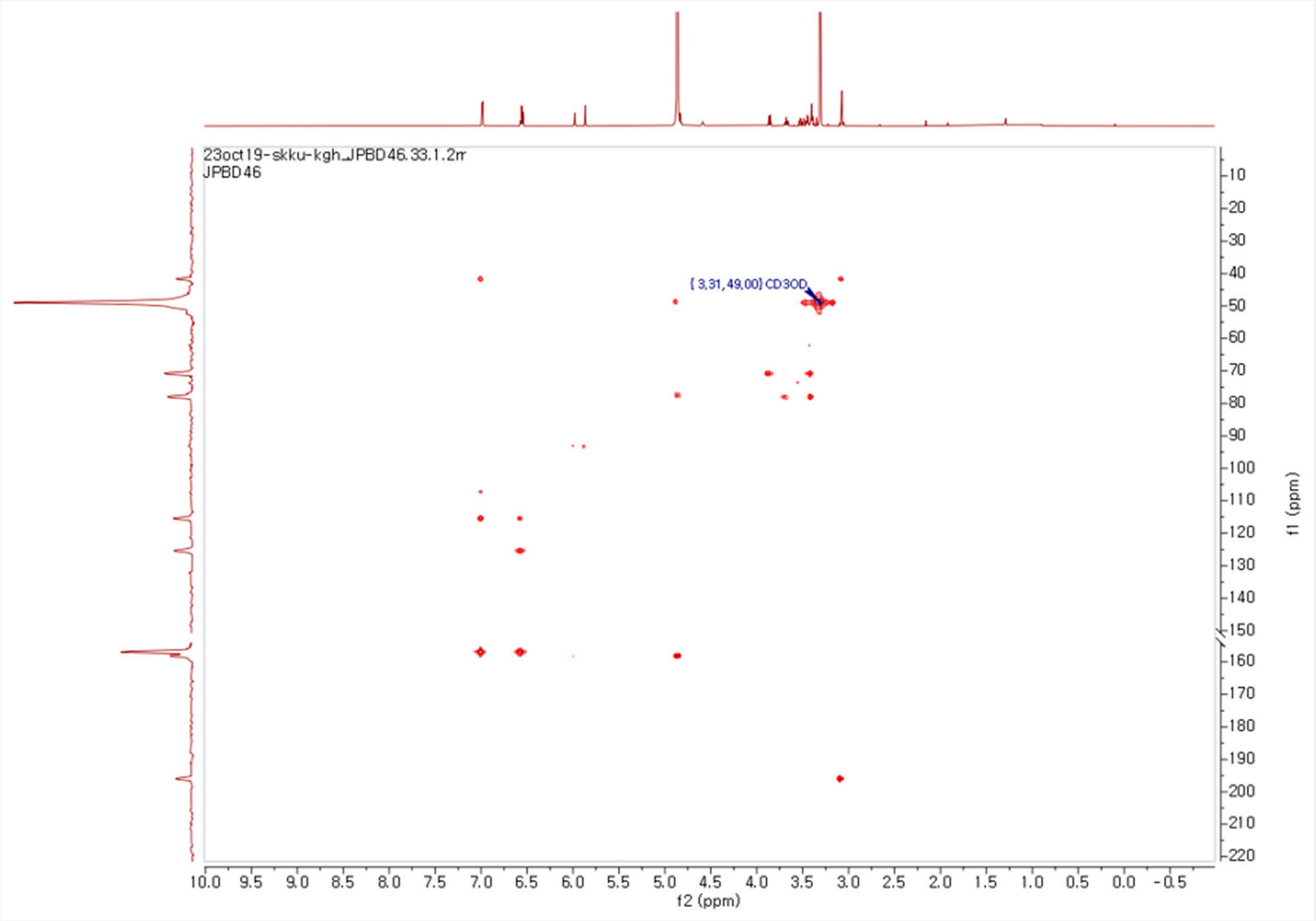

Figure S6. UV chromatogram of LC/MS, and UV and MS data for compound **2**

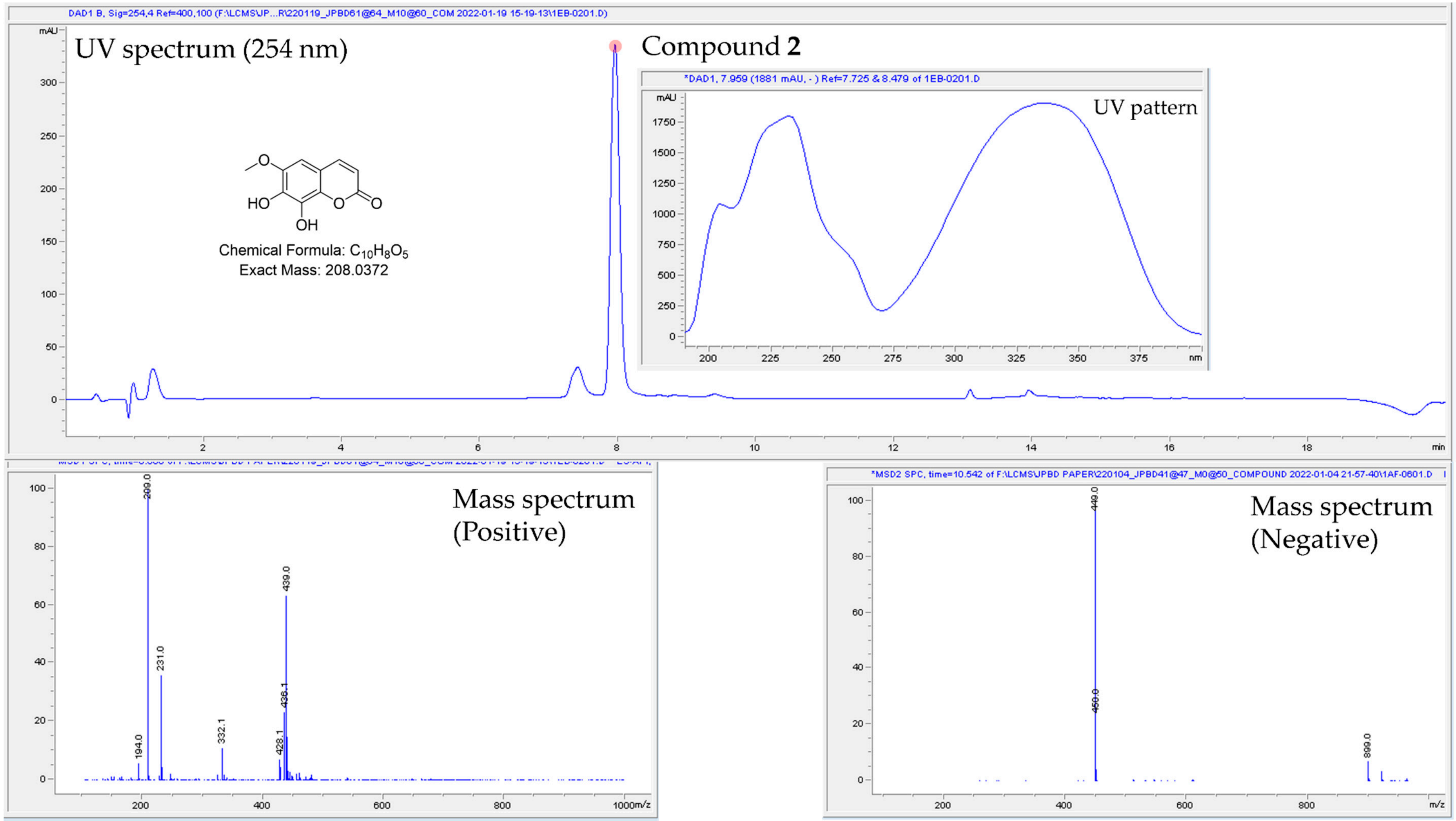

Figure S7. The ^{1}H NMR spectrum of **2** (CD_3OD, 850 MHz)

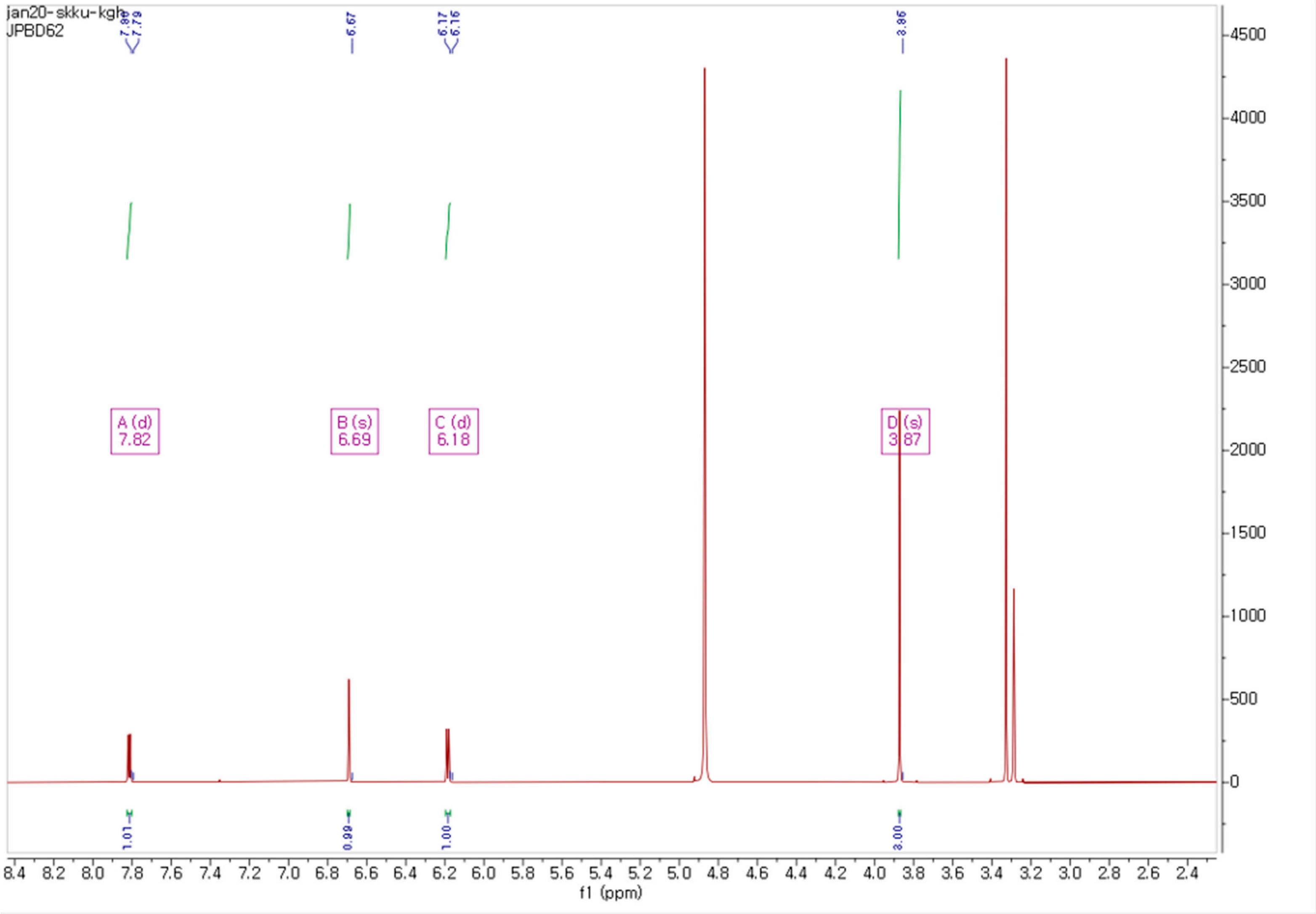

Figure S8. UV chromatogram of LC/MS, and UV and MS data for compound **3**

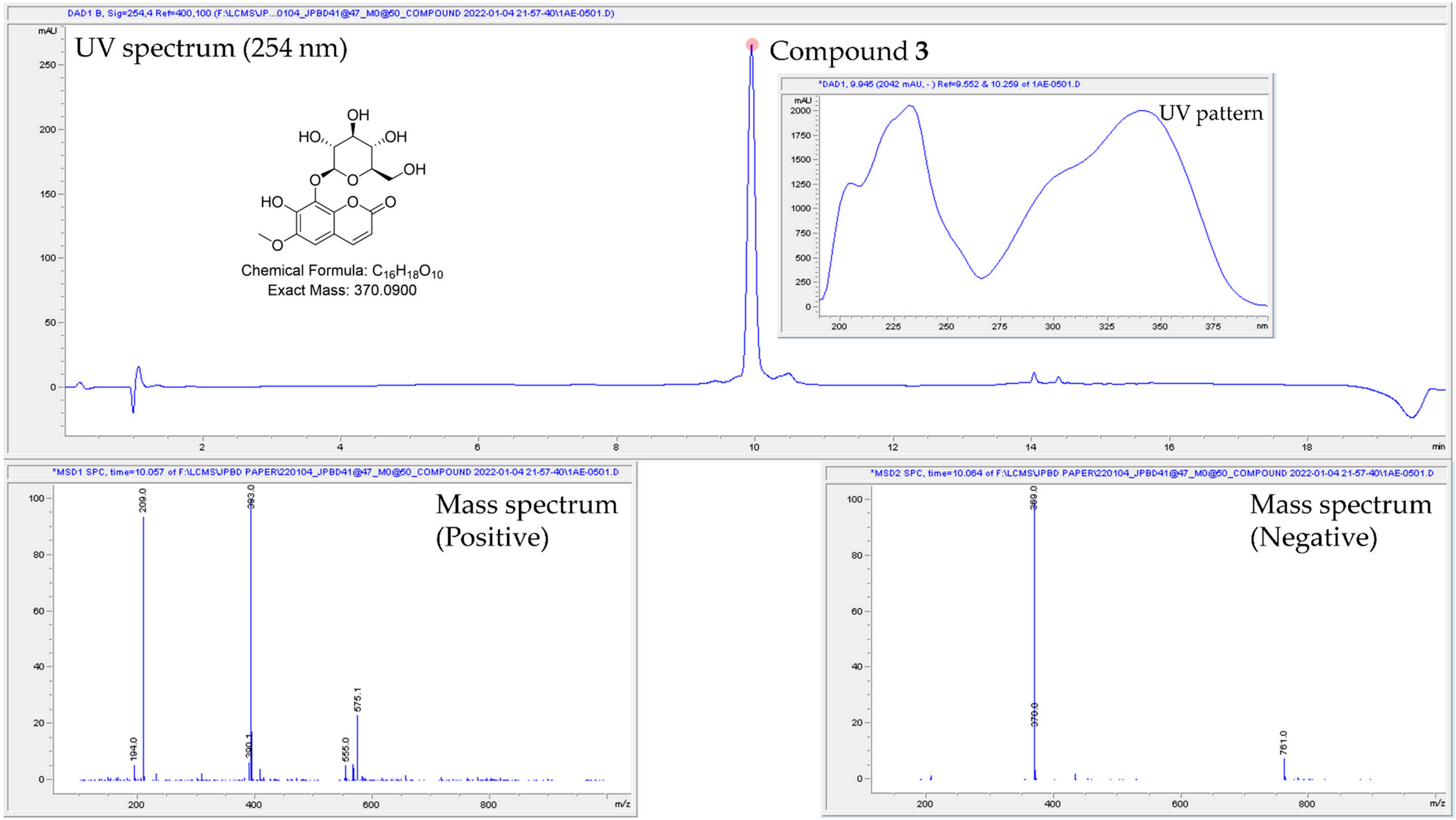

Figure S9. The ^{1}H NMR spectrum of **3** (CD_3OD, 850 MHz)

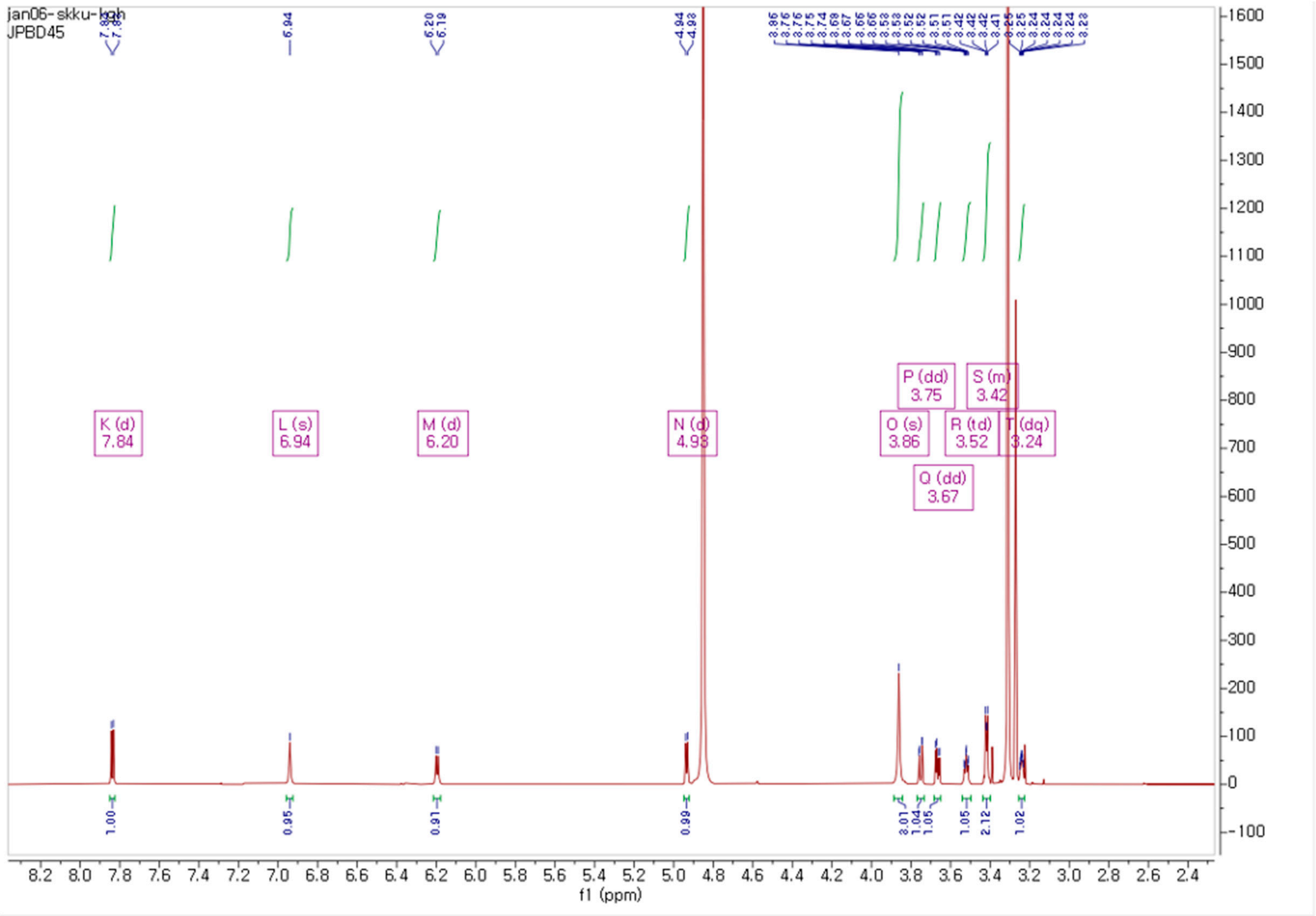

Figure S10. UV chromatogram of LC/MS, and UV and MS data for compound **4**

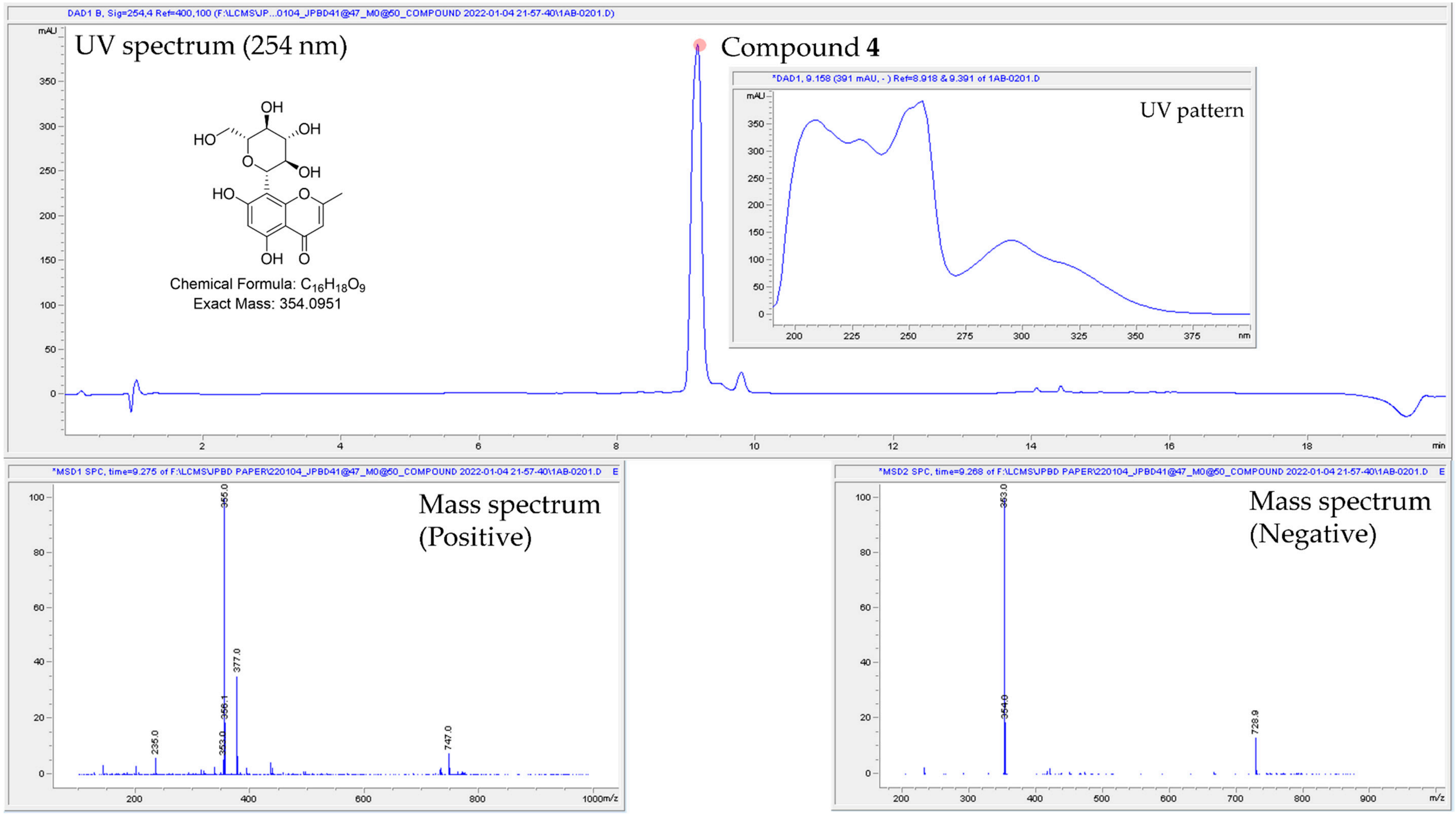

Figure S11. The ^{1}H NMR spectrum of **4** (CD_3OD, 850 MHz)

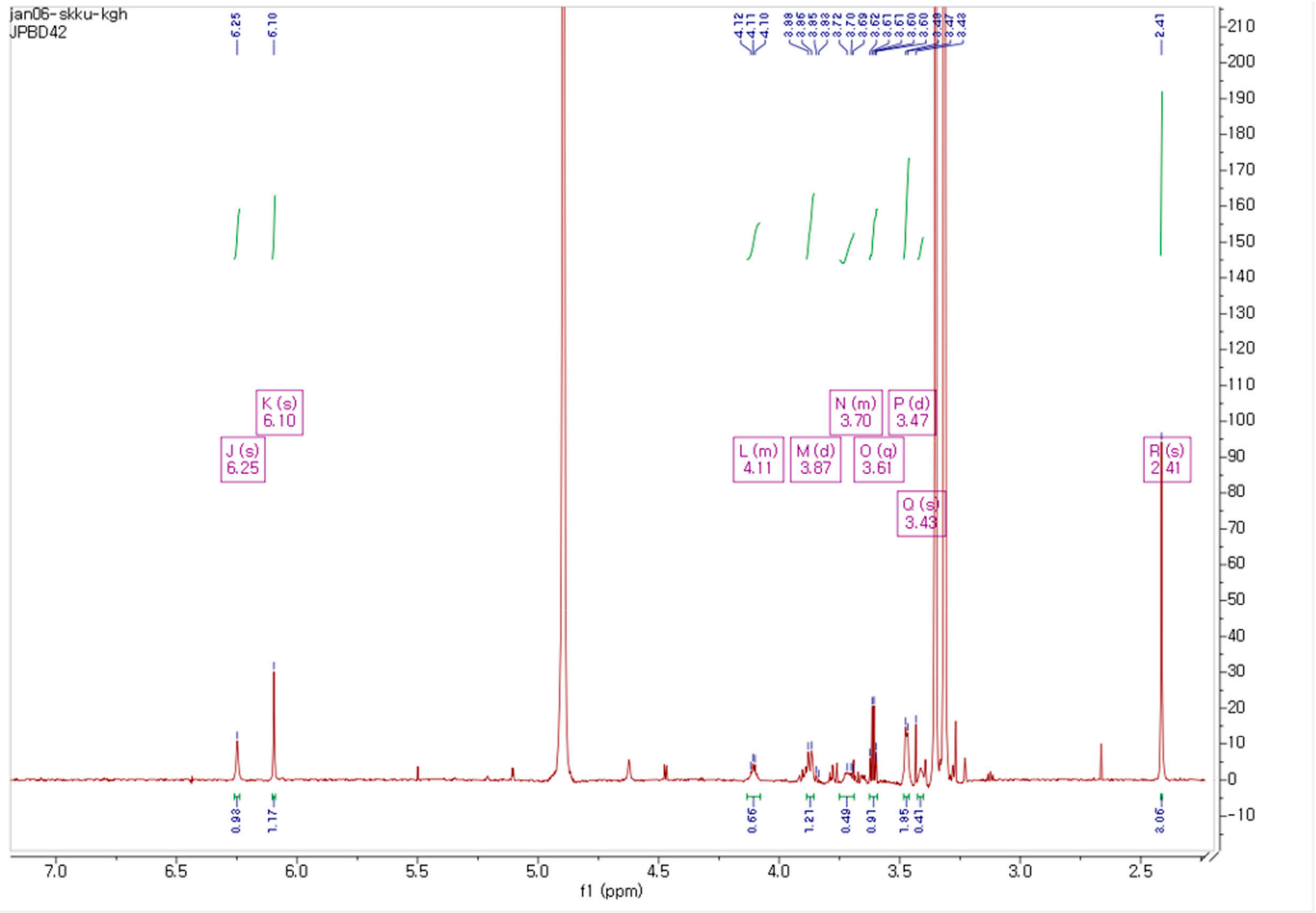

Figure S12. UV chromatogram of LC/MS, and UV and MS data for compound **5**

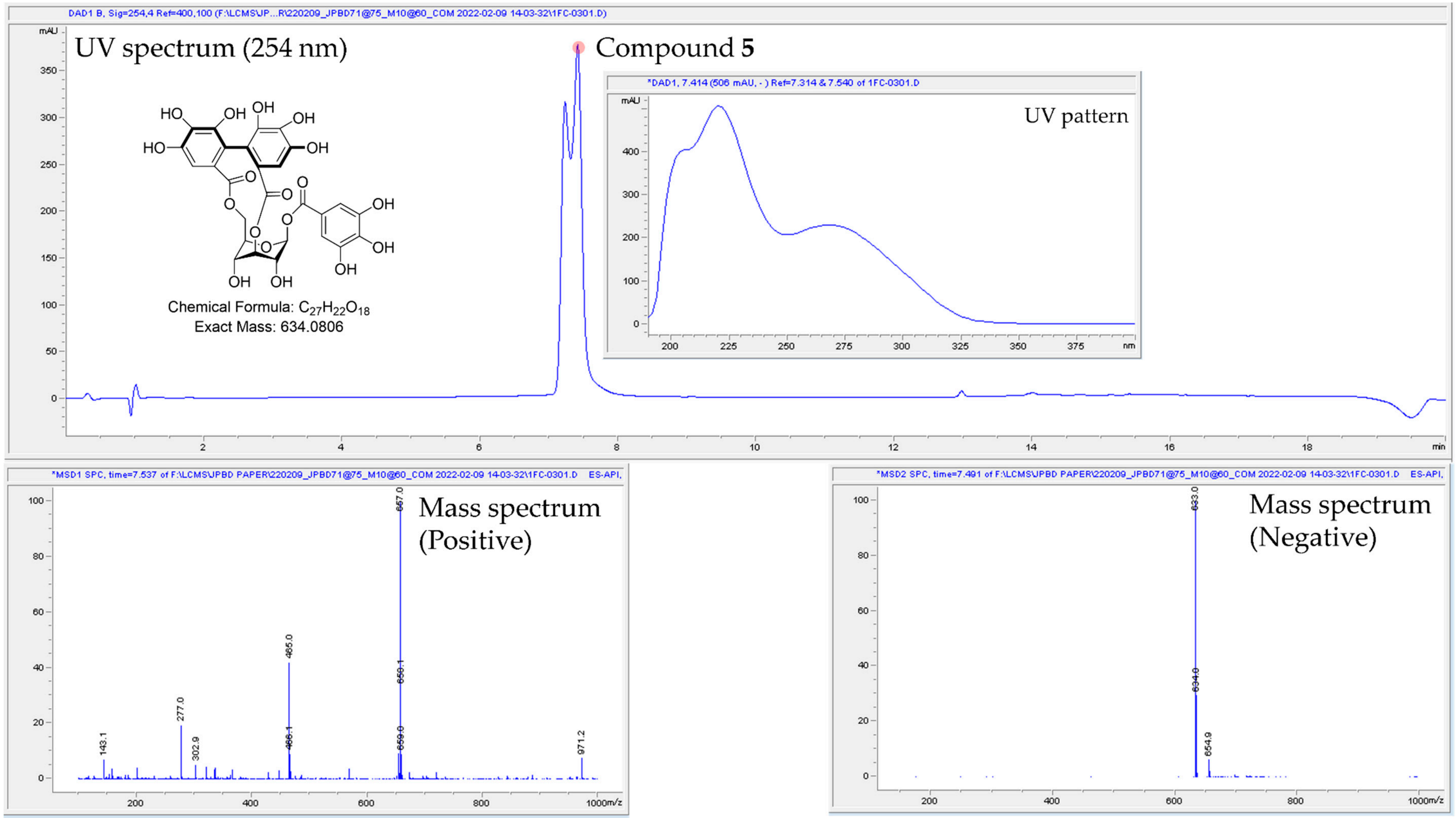

Figure S13. The ^{1}H NMR spectrum of **5** (CD_3OD, 850 MHz)

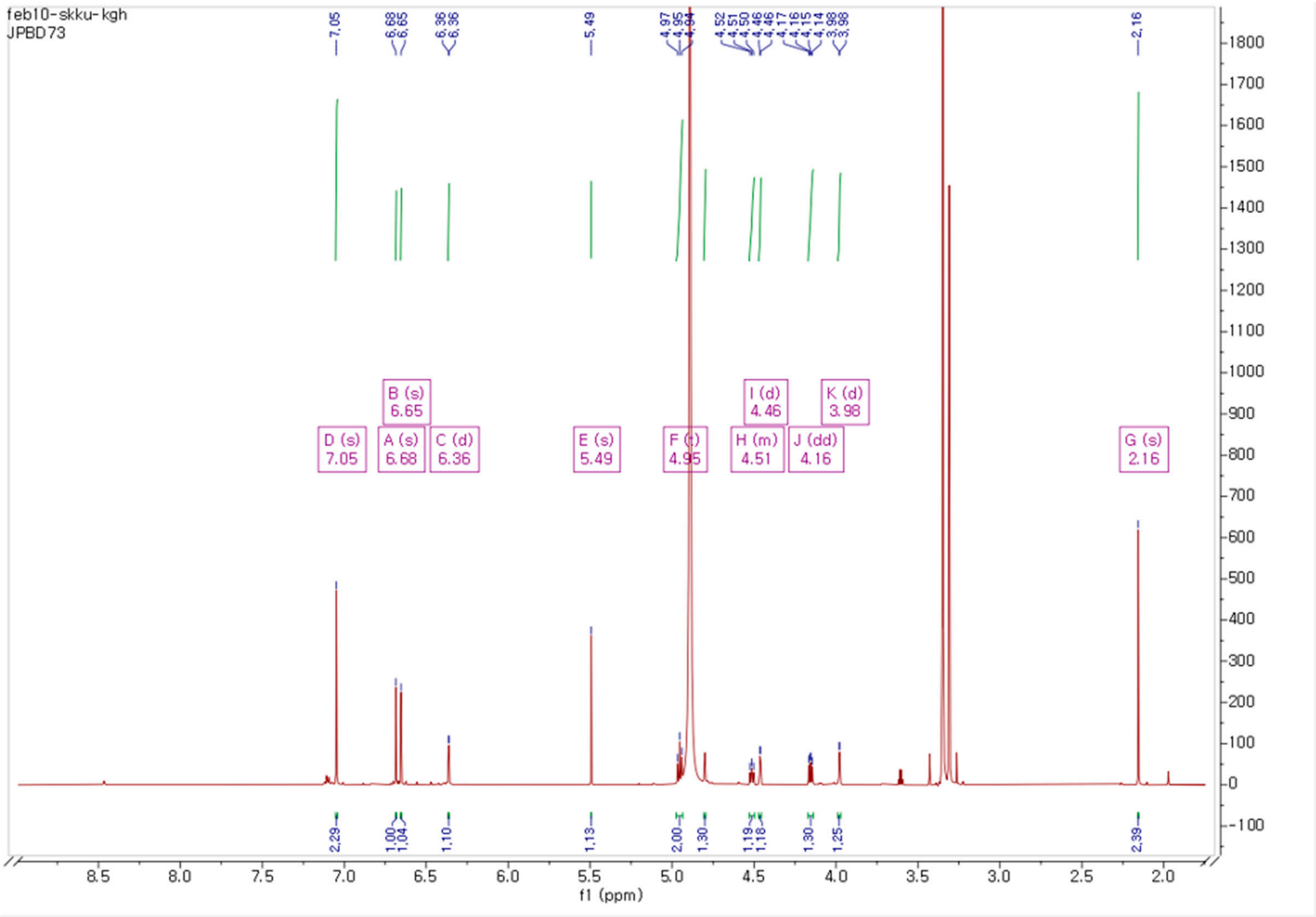

Supplement: Supplementary file 1 [file plants-12-03942-s001.zip › plants-2679735-supplementary.pdf]
